# Relationship between *GNAS1* T393C polymorphism and aseptic loosening after total hip arthroplasty

**DOI:** 10.1186/s40001-017-0271-z

**Published:** 2017-08-23

**Authors:** Patrick Stelmach, Max D. Kauther, Lena Fuest, Gina Kurscheid, Thorsten Gehrke, Stefanie Klenke, Marcus Jäger, Christian Wedemeyer, Hagen S. Bachmann

**Affiliations:** 1 Institute of Pharmacogenetics, University Hospital Essen, 45147 Essen, Germany; 2 Department of Orthopaedics and Trauma Surgery, University Hospital Essen, Essen, Germany; 3 Department of Joint Surgery, Helios ENDO-Klinik, Hamburg, Germany; 4 Department of Anaesthesiology and Intensive Care, University Hospital Essen, Essen, Germany; 5 Department of Health, School of Medicine, Institute of Pharmacology and Toxicology, Center for Biomedical Education and Research (ZBAF), Witten/Herdecke University, Witten, Germany

**Keywords:** GNAS1 T393C, Polymorphism, Aseptic loosening

## Abstract

**Background:**

Aseptic loosening is a main cause for revision surgery after total hip arthroplasty (THA) and there is no reliable marker for the early detection of patients at high risk. This study has been performed to validate association of the T393C polymorphism (rs7121) in the *GNAS1* gene, encoding for the alpha-subunit of heterotrimeric G-protein Gs, with risk for and time to aseptic loosening after THA, which has been demonstrated in our previous study.

**Methods:**

231 patients with primary THA and 234 patients suffering from aseptic loosening were genotyped for dependency on *GNAS1* genotypes and analyzed.

**Results:**

Genotyping revealed almost similar minor allele frequencies of 0.49 and 0.46, respectively. Consistently, genotype distributions of both groups were not significantly different (*p* = 0.572). Neither gender nor *GNAS1* genotype showed a statistically significant association with time to loosening (*p* = 0.501 and *p* = 0.840). Stratification by gender, as performed in our previous study, was not able to show a significant genotype-dependent difference in time (female *p* = 0.313; male *p* = 0.584) as well as median time to aseptic loosening (female *p* = 0.353; male *p* = 0.868).

**Conclusion:**

This study was not able to confirm the results of our preliminary study. An association of the *GNAS1* T393C polymorphisms with risk for and time to aseptic loosening after THA is unlikely.

## Background

The number of total hip arthroplasties (THA) will increase noticeably in the next years. For 2030, Kurtz et al. predicted a demand of about 600,000 THA in the United States [1]. Unfortunately, revision surgery after THA is often needed due to instability, infection as well as aseptic loosening [2]. This leads to a predicted doubling of the number of revision surgeries after THA by the year 2026 [1]. The identification of risk factors influencing time to aseptic loosening may result in a better outcome of patients with THA by application of specific prophylactic treatments prior or subsequent to surgery and may therefore attenuate the number of revisions and the predicted increasing financial burdens [3].

Over the last years, research focused mainly on the identification of clinical risk factors such as gender, body-mass-index (BMI), and age [4–6]. But some studies investigating the impact of genetic host factors on aseptic loosening have also been published [7, 8].

The *GNAS1* gene is located on chromosome 20q13.3 and encodes for the alpha-subunit of the heterotrimeric G-protein complex Gs. This G-protein complex interacts with G-protein-coupled receptors (GPCRs) and is an important molecular bottleneck in signal transduction pathways by increasing cellular second messenger cAMP level [9, 10]. Different studies demonstrated that Gαs alterations are causative for several bone diseases. The autosomal dominant disorder Albright’s hereditary osteodystrophy with reduced expression or function of Gαs leads to brachydactyly, short stature, and subcutaneous ossifications [11]. In McCune–Albright syndrome, an activating mutation of the *GNAS1* gene results in polyostotic fibrous dysplasia [12, 13]. Downregulation of *GNAS1* gene expression in osteoclasts by the antiretroviral drug tenofovir resulted in osteoclast dysfunction and clinical relevant bone density loss [14].

Exon 5 of the *GNAS1* gene harbors the common silent polymorphism T393C (rs7121) which was first characterized by Jia et al. [15]. They revealed a significant impact of this polymorphism on sympathetic signal transduction which was confirmed in further studies [16–18]. Furthermore, associations of this polymorphism with the course of other diseases, e.g., schizophrenia and different types of cancer have been reported [19–23]. However, other studies failed to show significant associations of this polymorphism [24, 25].

Recently, we investigated for the first time putative effects of the *GNAS1* T393C polymorphism on early aseptic loosening in a study comprising 57 patients after total hip arthroplasty [26]. There was no significant association between time and median time to aseptic loosening and *GNAS1* T393C genotypes. However, further analysis corrected for gender revealed that time to aseptic loosening was significantly longer in male patients. Therefore, additional analysis to investigate the effect of the *GNAS1* T393C polymorphism was performed and corrected for gender. The CC genotype was associated with significantly longer time and median time to aseptic loosening in male patients. In female patients, results were not completely consistent, but there was evidence that in contrast to male patients the TT genotype was associated with longer time and median time to aseptic loosening.

To date, no further analyses have been performed to validate these findings in independent studies. Therefore, we used a recently established independent cohort comprising 234 patients suffering from aseptic loosening and 231 patients with primary THA to prove the putative association of the *GNAS1* T393C polymorphism with aseptic loosening [27].

## Methods

### Patients

Our retrospective case–control study included 465 Western European patients of German ancestry operated at the Helios ENDO-Klinik Hamburg, Germany [27]. The control group consisted of 231 patients with primary THA without aseptic loosening and the case group consisted of 234 patients suffering from aseptic loosening after THA. Control and aseptic loosening patients were consecutively enrolled at the time of primary THA and revision surgery, respectively. The following inclusion and exclusion criteria were defined: inclusion criterion for the control group was primary THA due to primary osteoarthritis. Inclusion criteria for the case group were clinical, radiological, and intra-surgical diagnosis of aseptic loosening after THA due to primary osteoarthritis. Exclusion criteria for the case group were inflammatory diseases, treatment with immunosuppressant agents, traumatic loosening, any deep infection or the suspicion of implant infection. Exclusion of infection was carried out by microbiological swab analysis. The study was approved by the local Ethics Committee of the University Hospital Essen and performed according to the Declaration of Helsinki. Written informed consent was obtained from all patients on enrollment.

### Determination of GNAS1 T393C genotypes

DNA was obtained from whole blood or buccal swab using the QIAamp DNA blood mini kit (Qiagen, Hilden, Germany) following the manufacturer’s protocols. Genotypes of the T393C polymorphism (rs7121) were determined by PCR and restriction fragment length analysis as previously described [26]. Forward primer 5′-TGTGGCCGCCATGAGCAA-3′ and reverse primer 5′-TAAGGCCACACAAGTCGGGGT-3′ were used with the following PCR conditions: initial denaturation at 94 °C, followed by 38 cycles of DNA amplification at 94 °C for 45 s, 62 °C for 40 s, and 72 °C for 45 s. The obtained 145-bp PCR products were digested by BseGI (Fermentas, Germany) and separated on a 2.5% agarose gel. Completely restricted products of 73 and 72 bp represented the CC genotype and unrestricted products of 145 bp the TT genotype.

### Statistical analysis

We used the log-rank test and Kaplan–Meier plots to evaluate retrospectively the relationship between *GNAS1* genotypes, gender and time to loosening. The nonparametric Kruskal–Wallis test was performed in order to relate the median time to loosening to gender as well as genotype. The impact of age, BMI, and *GNAS1* T393C genotype as prognostic factors for time to aseptic loosening were analyzed by univariate and multivariate Cox regression models. From these Cox regression models, hazard ratios (HR) and 95% CI were calculated. For linear comparison of nonparametric variables, we performed a Jonckheere–Terpsta test. Continuous variables were compared by ANOVA and categorical variables using *GNAS1* genotypes were compared by contingency tables and Pearson’s *χ* ^2^ test. Differences were regarded as significant at *p* < 0.05. Statistical analyses were performed using SPSS 20.0 (SPSS, Chicago, IL, USA) and GraphPad Prism 6.0 (GraphPad Software, San Diego, CA). A public domain program was used to control for deviation from Hardy–Weinberg equilibrium [28]. Power was calculated using the publically available software PS 3.1.2 [29, 30].

## Results

### GNAS1 T393C genotype and clinical characteristics

Clinical characteristics and genotype distribution in patients with primary THA and patients with aseptic loosening are given in Table 1. Age at implantation and replantation, BMI, gender, first stem with or without cement, and first cup with or without cement are listed. The *GNAS1* T393C genotype distribution of the 231 patients with primary THA was TT in 52 patients, TC in 122 patients, and CC in 57 patients. *GNAS1* T393C genotype distribution in 234 patients suffering from aseptic loosening after THA was 44 TT genotype carriers, 126 heterozygous patients and 64 carried the CC genotype. Both genotype distributions were not significantly different from Hardy–Weinberg equilibrium (primary THA: *p* = 0.388; aseptic loosening: *p* = 0.194). The genotype distribution was neither significantly different between the two groups (*p* = 0.572) nor to another already published control group consisted of 820 healthy German individuals of Caucasian ancestry (*p* = 0.389) [31]. Furthermore, the analysis of the clinical characteristics gender, age at enrollment, and BMI showed no statistically significant differences between the two groups (Primary THA, age at implantation: 64.54 ± 10.9 years vs. aseptic loosening, age at replantation: 65.34 ± 12.4 years, *p* = 0.461; primary THA, BMI: 27.31 ± 4.5 kg/m^2^ vs. aseptic loosening: 26.94 ± 5.9 kg/m^2^, *p* = 0.448; primary THA, gender: 65.4% female vs. aseptic loosening: 70.5% female, *p* = 0.235). We analyzed whether there was a correlation of these clinical variables of the control and the study group with *GNAS1* T393C genotype. However, no statistically significant correlation was found (Table 1).

**Table 1:** Clinical characteristics and genotype distribution in patients with primary THA and patients with aseptic loosening

	All	*GNAS1* T393C genotype			*p* value
		TT	TC	CC	
Primary THA					
*n* (%)	231	52 (22.5)	122 (52.8)	57 (24.7)	
Age at implantation (years)	64.54 ± 10.9	62.12 ± 11.3	65.77 ± 10.0	64.12 ± 12.0	0.136
Gender					
Female (%)	151 (65.4)	39 (25.8)	80 (53.0)	32 (21.2)	0.118
Male (%)	80 (34.6)	13 (16.3)	42 (52.5)	25 (31.3)	
BMI (kg/m^2^)	27.31 ± 4.5	26.95 ± 4.6	27.64 ± 4.3	27.00 ± 4.9	0.556
Aseptic loosening					
*n* (%)	234	44 (18.8)	126 (53.8)	64 (27.4)	
Age at implantation (years)	52.80 ± 12.8	51.44 ± 13.6	53.08 ± 13.1	53.22 ± 11.6	0.735
Age at replantation (years)	65.34 ± 12.4	63.20 ± 12.6	65.53 ± 12.7	66.46 ± 11.9	0.399
Gender					
Female (%)	165 (70.5)	31 (18.8)	87 (52.7)	47 (28.5)	0.821
Male (%)	69 (29.5)	13 (18.8)	39 (56.5)	17 (24.6)	
BMI (kg/m^2^)	26.94 ± 5.9	27.15 ± 4.8	27.13 ± 7.0	26.45 ± 3.9	0.734
First cup with cement (*n* = 212)					
No	73 (34.4)	15 (20.5)	37 (50.7)	21 (28.8)	0.749
Yes	139 (65.6)	26 (18.7)	78 (56.1)	35 (25.2)	
First stem with cement (*n* = 213)					
No	75 (35.2)	14 (18.7)	42 (56.0)	19 (25.3)	0.946
Yes	138 (64.8)	27 (19.6)	74 (53.6)	37 (26.8)	

Data are numbers with percentages given in brackets and numbers with standard deviation, respectively. Categorical variables were analyzed by *χ* ^2^ statistics. *p* values were calculated using ANOVA for continuous variables

### GNAS1 T393C genotype and time to aseptic loosening

**Fig. 1:**
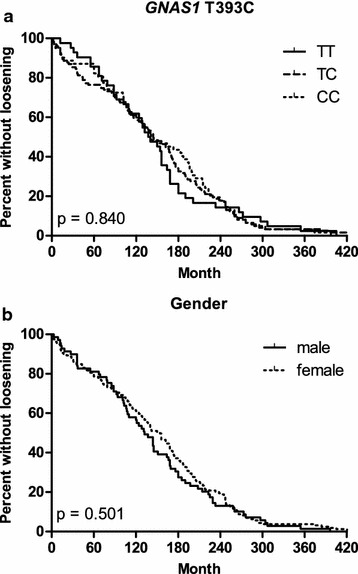
Time to aseptic loosening depending on *GNAS1* T393C genotype and gender. Time to aseptic loosening based on Kaplan–Meier-curves for 234 patients with aseptic loosening. **a** Patients based on *GNAS1* T393C TT, TC and CC genotype. **b** Patients based on gender

In our previous study, a gender-dependent association of *GNAS1* T393C polymorphism with aseptic loosening was found. Therefore, time and median time to aseptic loosening were analyzed for correlation with *GNAS1* T393C genotype and gender using Kaplan–Meier survival curves and Kruskal–Wallis test in this study (Fig. 1; Table 2). Time and median time to aseptic loosening after THA were neither significantly associated with *GNAS1* T393C genotype nor with gender (Fig. 1a, b; Table 2). Median time to aseptic loosening was 142 months (range 1–431) for all 234 patients, for *GNAS1* T393C TT homozygous patients 134.5 months (range 1–405), for *GNAS1* T393C CC homozygous patients 141.5 months (range 2–397) and for *GNAS1* T393C TC heterozygous patients 144 months (range 1–431). Median time to aseptic loosening was 152 months (range 1–431) for female patients and 132 months (range 3–396) for male patients.

**Table 2:** Median time to aseptic loosening according to gender and *GNAS1* genotype

	All	Gender		*p* value
		Female	Male	
*n* (%)	234	165 (70.5)	69 (29.5)	
MTAL, months (range)	142 (1–431)	152 (1–431)	132 (3–396)	0.576

	All	*GNAS1* T393C genotype			*p* value
		TT	TC	CC	
*n* (%)	234	44 (18.8)	126 (53.8)	64 (27.4)	
MTAL, months (range)	142 (1–431)	134.5 (1–405)	144 (1–431)	141.5 (2–397)	0.667
Female					
*n* (%)	165	31 (18.8)	87 (52.7)	47 (28.5)	
MTAL, months (range)	152 (1–431)	137 (1–405)	159 (1–431)	173 (2–397)	0.353*
Male					
*n* (%)	69	13 (18.8)	39 (56.5)	17 (24.6)	
MTAL, months (range)	132 (3–396)	128 (27–354)	144 (3–396)	108 (12–304)	0.868

Data are numbers with percentages given in brackets and medians with ranges given in brackets, respectively. *p* values were calculated using Kruskal–Wallis test for nonparametric variables. *Jonckheere–Terpstra test for linear comparison of nonparametric variables *p* = 0.160

*MTAL* median time to aseptic loosening

**Fig. 2:**
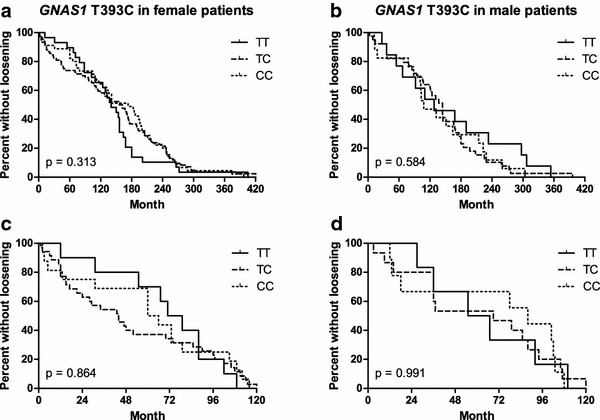
Time to aseptic loosening depending on *GNAS1* T393C genotype stratified by gender. Time to aseptic loosening based on Kaplan–Meier curves for 165 female and 69 male patients, respectively. **a**, **b** All female and male patients, based on *GNAS1* T393C genotype. **c**, **d** Female and male patients with aseptic loosening within 10 years after THA based on *GNAS1* T393C genotype

Next we investigated the putative correlation between *GNAS1* T393C genotypes and aseptic loosening stratified by gender. No significant correlation between time (*p* = 0.313 for females and *p* = 0.584 for males) as well as median time (*p* = 0.353 for females and *p* = 0.868 for males) to aseptic loosening and *GNAS1* T393C genotype could be detected for males or females (Fig. 2a, b; Table 2). In our previous study, all 57 patients suffered from early aseptic loosening within 10 years after THA. Therefore, in a third step, only female and male patients with aseptic loosening within 10 years after THA were analyzed. However, no statistically significant correlation between time (*p* = 0.864 for females and *p* = 0.991 for males) to aseptic loosening and *GNAS1* T393C genotype could be detected (Fig. 2c, d). Under the assumption of expected hazard ratios comparable to those of our preliminary study [26], power analysis revealed a power of at least 80% for these tests.

In line with the results from Kaplan–Meier curves and Kruskal–Wallis test, female and male patients showed no significant differences in time to aseptic loosening between single genotypes despite correcting for the variables age and BMI in univariate and also in multivariate analysis (Table 3).

**Table 3:** Factors influencing the time to aseptic loosening by univariate and multivariate Cox-regression analysis

Variable	Multivariate analysis											
	All				Female				Male			
	p*	*p*	HR	95% CI	p*	*p*	HR	95% CI	p*	*p*	HR	95% CI
T393C												
TT			1^a^				1^a^				1^a^	
TC	0.648	0.427	0.868	0.61–1.23	0.166	0.079	0.685	0.45–1.05	0.354	0.329	1.407	0.71–2.79
CC	0.564	0.342	0.828	0.56–1.22	0.172	0.124	0.695	0.44–1.11	0.344	0.572	1.245	0.58–2.66
Age	<0.001	<0.001	1.037	1.02–1.05	<0.001	<0.001	1.036	1.02–1.05	0.003	0.002	1.039	1.01–1.06
BMI	0.158	0.030	1.020	1.00–1.04	0.286	0.068	1.018	0.99–1.04	0.192	0.176	1.051	0.98–1.13

* Univariate analysis

^a^Reference group

## Discussion

Aseptic loosening is a common problem after THA and will cause increased financial burdens in the next years [3]. Unfortunately, the number of replacements in each individual patient is limited [32]. Therefore, replacement due to aseptic loosening is a clinically relevant problem. Thus, the identification of a prognostic marker of aseptic loosening would be very useful. Patients at high risk for aseptic loosening after THA could be identified before surgery and might benefit for example from pronounced weight reduction or special prophylactic treatments. The advantages of a genetic host factor as a prognostic marker would be an easy determination, realizable prior to surgery. In recent years, some promising polymorphism in different genes such as the Matrix metalloprotease 1 [33], the Tissue Inhibitor of Metalloproteinase 1 [34], or BCL2 [27] were identified and their impact on risk for and time to aseptic loosening were investigated. Despite significant associations of these polymorphisms with risk for and time to aseptic loosening, up to date, none of these polymorphisms is determined routinely. In this study, we investigated the impact of the single-nucleotide polymorphism T393C in the *GNAS1* gene locus on time and median time to aseptic loosening. Time and median time to aseptic loosening after THA were neither significantly associated with *GNAS1* T393C genotypes nor with gender. Stratification by gender revealed no significant association with *GNAS1* T393C genotypes. These results are obviously not in line with the results of our previous study which revealed a significant gender-dependent association with aseptic loosening after THA and after stratification by gender a significant association of *GNAS1* T393C polymorphism with aseptic loosening after THA [26].

The previous study was preliminary because of the limited number of participants comprising only 57 patients suffering from aseptic loosening. Therefore, we used a recently established cohort to perform this independent replication study. Comparison of hazard ratio 95% confidence intervals of both studies shows overlapping intervals indicating that results do not exclude each other. Furthermore, we can assume that both cohorts might belong to the same basic population and no fulminant structural bias was apparent in one of our studies. Because this study was sufficiently powered to falsify the previous results, we assume a type I error led to the significant findings of our previous study. Nevertheless, it cannot be completely ruled out that in an even larger study population a significant effect could be demonstrated. But due to our results this effect would be small and the question remains if such a weak association is good enough to be useful as a prognostic clinical marker. Furthermore, it should be mentioned that also other known or unknown risk factors, e.g., the type of implants that have been used, the quality of the cement technique and the surgical experience may act as potentially influencing factors on time to aseptic loosening. In future studies, these factors should be considered in order to exclude additional potential confounders. However, if it would be possible to verify a couple of genetic markers, each with a small impact, development of genetic host factor panels might be an option to personalize the treatment in future. Finally, despite the identification of some polymorphisms in different genes and their association to risk for and time to aseptic loosening [27, 33, 34], other, not yet identified polymorphisms may have an even stronger effect on aseptic loosening. Therefore, we are convinced that further research on genetic host factors is a promising tool to discover new prognostic markers in aseptic loosening after THA.

## Conclusion

Our study emphasized that an association of the polymorphism T393C located in the *GNAS1* gene locus with risk for and time to aseptic loosening is unlikely. Further independent and prospective studies should be undertaken to rule out the outstanding (genetic) key reasons for aseptic loosening after THA. Yet unstudied polymorphisms might play a major role in the pathogenesis of aseptic loosening.

## Abbreviations

THA: total hip arthroplasty
BMI: body-mass-index
GPCRs: G-protein-coupled receptors
cAMP: cyclic adenosine monophosphate
